# Supplementary material for: COZOID: contact zone identifier for visual analysis of protein-protein interactions
Source: BMC Bioinformatics. 2018 Apr 6;19:125. doi: 10.1186/s12859-018-2113-6 (PMC5889581; doi:10.1186/s12859-018-2113-6)
Supplement: Supplementary file 2 — Software build. Executable binary file of the software tool. (ZIP 118,784 kb) [file 12859_2018_2113_MOESM2_ESM.zip › cozoid/platform/docs/testng-6.5.1-javadoc.zip!/org/testng/IAnnotationTransformer.html]

IAnnotationTransformer (TestNG)


---


|  |  |  |
| --- | --- | --- |
| **Overview** \| **Package** \| **Class** \| **Tree** \| **Deprecated** \| **Index** \| **Help** | |  |
| **PREV CLASS**   **NEXT CLASS** | **FRAMES**    **NO FRAMES**     **All Classes** |  |
| SUMMARY: NESTED \| FIELD \| CONSTR \| METHOD | DETAIL: FIELD \| CONSTR \| METHOD |  |


---


## org.testng Interface IAnnotationTransformer

**All Superinterfaces:**: ITestNGListener

**All Known Subinterfaces:**: IAnnotationTransformer2

---

``` public interface IAnnotationTransformer extends ITestNGListener ```

---

| **Method Summary** | |
| --- | --- |
| `void` | `transform(ITestAnnotation annotation, java.lang.Class testClass, java.lang.reflect.Constructor testConstructor, java.lang.reflect.Method testMethod)`             This method will be invoked by TestNG to give you a chance to modify a TestNG annotation read from your test classes. |

**Method Detail**

### transform

```
void transform(ITestAnnotation annotation,
               java.lang.Class testClass,
               java.lang.reflect.Constructor testConstructor,
               java.lang.reflect.Method testMethod)
```

:   This method will be invoked by TestNG to give you a chance
    to modify a TestNG annotation read from your test classes.
    You can change the values you need by calling any of the
    setters on the ITest interface.
    Note that only one of the three parameters testClass,
    testConstructor and testMethod will be non-null.

    :   **Parameters:**: `annotation` - The annotation that was read from your test class.: `testClass` - If the annotation was found on a class, this parameter represents this class (null otherwise).: `testConstructor` - If the annotation was found on a constructor, this parameter represents this constructor (null otherwise).: `testMethod` - If the annotation was found on a method, this parameter represents this method (null otherwise).


---


|  |  |  |
| --- | --- | --- |
| **Overview** \| **Package** \| **Class** \| **Tree** \| **Deprecated** \| **Index** \| **Help** | |  |
| **PREV CLASS**   **NEXT CLASS** | **FRAMES**    **NO FRAMES**     **All Classes** |  |
| SUMMARY: NESTED \| FIELD \| CONSTR \| METHOD | DETAIL: FIELD \| CONSTR \| METHOD |  |


---
